# Short Exon Detection via Wavelet Transform Modulus Maxima

**DOI:** 10.1371/journal.pone.0163088

**Published:** 2016-09-16

**Authors:** Xiaolei Zhang, Zhiwei Shen, Guishan Zhang, Yuanyu Shen, Miaomiao Chen, Jiaxiang Zhao, Renhua Wu

**Affiliations:** 1 Shantou University Medical College, Shantou, P.R. China; 2 Department of Radiology, Second Affiliated Hospital of Shantou University Medical College, Shantou, P.R. China; 3 College of Engineering, Shantou University, Shantou, P.R. China; 4 College of Electronic Information and Optical Engineering, Nankai University, Tianjin, P.R. China; National Cheng Kung University, TAIWAN

## Abstract

The detection of short exons is a challenging open problem in the field of bioinformatics. Due to the fact that the weakness of existing model-independent methods lies in their inability to reliably detect small exons, a model-independent method based on the singularity detection with wavelet transform modulus maxima has been developed for detecting short coding sequences (exons) in eukaryotic DNA sequences. In the analysis of our method, the local maxima can capture and characterize singularities of short exons, which helps to yield significant patterns that are rarely observed with the traditional methods. In order to get some information about singularities on the differences between the exon signal and the background noise, the noise level is estimated by filtering the genomic sequence through a notch filter. Meanwhile, a fast method based on a piecewise cubic Hermite interpolating polynomial is applied to reconstruct the wavelet coefficients for improving the computational efficiency. In addition, the output measure of a paired-numerical representation calculated in both forward and reverse directions is used to incorporate a useful DNA structural property. The performances of our approach and other techniques are evaluated on two benchmark data sets. Experimental results demonstrate that the proposed method outperforms all assessed model-independent methods for detecting short exons in terms of evaluation metrics.

## 1 Introduction

**Fig 1:**
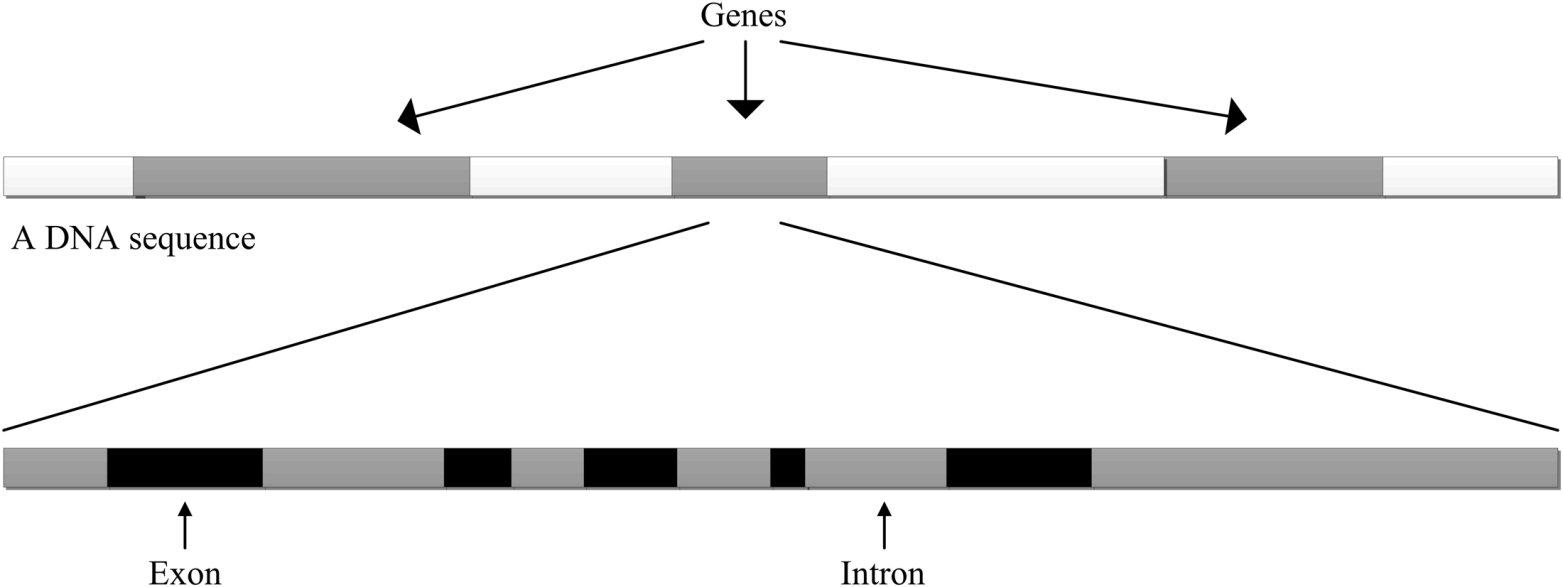
Organization of eukaryotic genes.

As an initial step in the analysis of eukaryotic genome sequences, detecting exons would lead to a good understanding of the structure and function of a protein that is synthesized by these exons [1, 2]. Unlike prokaryotes, eukaryotic genes are further divided into relatively small exons called protein coding regions and the introns called non-coding regions, as shown in Fig 1. In the past twenty years or so, many algorithms have been proposed for exon detection and good detection rate has been achieved in the recognition of exon and intron regions [1–33]. But despite the efforts spent, it is still an open question whether the strengths of the statistical features are sufficient to identify short exons. Typically, human exons are much shorter in length (137 base pairs (bp) in average) [6]. So the task for accurate and reliable methods to automatically determine the lengths and locations of short exons still needs to be solved today [2, 8–15, 32–33]. In addition, accurate location of short exons in genomes can help to design drugs and cure diseases. For example, some short exons of BRCA1 gene are related to ovarian and breast cancer [34–37]. Therefore, it is essential to develop an efficient technique for detecting the short exons of eukaryotic DNA sequences.

The detection of exons suggested in the literature can be classified into two categories, model-dependent methods and model-independent methods [38]. Model-dependent methods employ previously known genomic information or learning models to train the classifiers in the design of analysis stage [3–11], which means these techniques tend to be more precise. However, the issue of detecting short exons in eukaryotic genomes by using these methods still remain to be solved. Because on the one hand, assessing the coding potential of short sequences is not self-evident as intrinsic signals are harder to detect with shorter sequence lengths [11]; and on the other hand, methods such as Markov models rely on more sequences that contain short exons into the training sets [12]. Further, training datasets have limitations in dealing with the next-generation genome sequencing projects and can attach ascertainment bias to unknown genes or exons with a typical organization or structural components [10, 31]. By contrast, the methods designed to detect universal and statistical features of exons are model-independent as they do not assume any a prior information to train models or estimate parameters [38]. Model-independent methods for short exons detection, is considerably more challenging for some reasons. First, it is difficult to reveal the underlying property in the data [14]. For example, the statistic features obtained from short exons may be unreliable and the performance for short exon detection may be poor. Second, sequencing and frame shift errors such as insertions and deletions can also vitiate the techniques when the exons are short [15]. Nevertheless, model-independent methods may be adaptive enough to analyze the novel sequences when prior genomic information or training dataset is unavailable from the existing databases [2, 26–27, 30–31].

This paper is aimed at improving the detection accuracy of short exons in model-independent domain. It is well-known that the exons exhibit three-base periodicity (TBP), which is absent in other regions such as intergenic and intron regions in eukaryotes [39–43]. Model-independent methods based on digital signal processing (DSP) techniques and TBP are built upon the phenomenon that exon regions have a prominent power spectrum peak at frequency *f* = 1/3 [16–17]. During recent years, a large number of model-independent methods have been proposed to detect exons [1, 2, 16–31]. The detecting techniques which rely on discrete Fourier transform (DFT) include the spectral content measure [16] and its various improved versions [17–21]. The DFT-based methods have been the basis of several techniques in many ways. Vaidyanathan and Yoon [22] utilized anti-notch filters to solve the same problem. Yin and Yau [25] developed a novel exon detection algorithm, known as exon prediction by nucleotide distribution (EPND) to investigate the relationship between TBP and nucleotide distributions. Mena-Chalco et al. [26] introduced a method based on modified Gabor-wavelet transform (MGWT) for improving exon detection. This technique outperforms the sliding window approaches with respect to detection accuracy. Zhang and Yan [27] combined DNA structural profiles and empirical mode decomposition to improve short exon detection. This novel method named as the fast Fourier transform plus empirical mode decomposition (FFTEMD) provided a pictorial view of spectrum analysis of non-stationary signal. Recently, Marhon and Kremer [31] proposed the wide-range wavelet window (WRWW) method for extracting exon components. The WRWW can adapt its width to accommodate the change in the window length and it outperforms all assessed model-independent methods with relatively short and long exons.

To perform accurate detection of short exons, a natural solution of the problem is to extract and enhance their weak features embedded in background noise of intron regions. Existing methods provide a description of the overall regularity of exons, but they are not well adapted for finding the location and spatial distribution of singularities represented by short exons. This is the major motivation to study the singular measure and its application to short exon detection. As a generalized multifractal formalism for fractal functions and singular measures, the wavelet transform modulus maxima (WTMM) [44–45] has proved its success in studying the long-range correlation properties of genomic DNA sequences [45–48] and analyzing the strand compositional asymmetry profiles in relation to transcription and replication [49–52], by providing “oscillating boxes” to get rid of possible smooth behavior that may either mask the singularities or perturb the estimation of their strength. A comprehensive review of the research of wavelet-based multi-scale signal processing and WTMM on genomic information has been summarized in [53].

In this work, we will follow the WTMM-based strategy inspired from the one previously used in the analysis of genomic information [45–53] to detect short exons. Firstly, the paired-numerical representation is employed to incorporate a useful structural property and reduce the computational cost. We apply this numerical representation to construct the nucleotide distribution sequence. In this design, the statistical properties in the three reading frames is extracted from DNA sequences to differentiate between exon and intron regions. Then the nucleotide distribution sequence is used to calculated the TBP spectrum by an optimized WTMM-based method. Finally, to reflect a reality of the structure of double helix DNA, the output values are calculated along the forward and reverse directions. Case studies indicate that the proposed method improves detection accuracy of exons in comparison with existing methods and exceeds its counterparts in the ability to detect short exons.

## 2 Methods

### 2.1 Related Works

#### 2.1.1 Numerical representation of a DNA sequence

A symbolic DNA sequence is composed of four different nucleotides (or bases) named Adenine (A), Thymine (T), Cytosine (C) and Guanine (G). In order to employ DSP tools on a DNA sequence, the first step in the process is to convert symbolic DNA sequences into numerical sequences. In this paper, we introduce the paired-numerical representation [21]. The paired representation exploits the property of the frequency distribution of nucleotides *A−T* and *C−G*, which is based on the fact that intron regions are rich in nucleotides *A* and *T* while exon regions are rich in nucleotides *C* and *G*. This representation could provide more differentiation between exon and intron regions when the TBP is investigated. This paired representation scheme could decrease the computation time compared to three-(Z-curve) [9] and four-(Voss) [42] sequence representations. Using this paired-numerical representation, the presence of nucleotide *A* or *T* at a particular base pair position is denoted by 1, and the absence of it is denoted by 0. Similarly, the existence of nucleotide *C* or *G* in a relative base pair position is represented by -1 and the absence of the nucleotide is represented by 0. An example of this representation scheme of a DNA sequence is presented in Table 1.

**Table 1:** An example of the paired-numerical representation scheme of a DNA sequence.

	A	C	C	A	G	G	T	A	…
*u*_*A*−*T*_	1	0	0	1	0	0	1	1	…
*u*_*C*−*G*_	0	-1	-1	0	-1	-1	0	0	…

#### 2.1.2 Calculating the TBP spectrum from occurrence frequencies of nucleotides

The nucleotide distribution in the three reading frames incorporates the reality of proteins' prefer specific amino acid compositions [39]. There exists a relationship between the occurrence frequencies of a nucleotide in the three codon positions and the TBP spectrum [24]. In this section, we introduce an efficient way to compute the TBP spectrum by calculating the nucleotide occurrence frequencies in the three codon positions. This approach can reduce the computing time significantly by comparing with Fourier transform.

Let *u*_*x*_(*x* ∈ {*A*−*T*, *C*−*G*}) be a paired-numerical sequence of length *N*, the DFT power spectrum of a length-*N* block of *u*_*x*_ is defined as follows:

$$S_{x}[k]={\left|\sum_{n=0}^{N-1}u_{x}[n]e^{-i2\pi nk/N}\right|}^{2},\;k=0,1,\ldots ,N-1,$$ (1)

where *i*^2^ = −1. The TBP in exon regions of a DNA sequence suggests that the DFT power spectrum *S*_*x*_[*k*] corresponding to *k* = *N*/3 (where *N* is chosen to be a multiple of 3) in each DFT sequence should be large [16, 17]. By sliding a window across the sequence, we can determine the TBP value at each position.

Let *f*_*x*_(0), *f*_*x*_(1), *f*_*x*_(2) (*x* ∈ {*A*−*T*, *C*−*G*}) denote the occurrence frequencies of a nucleotide in the first, the second and the third codon position of *u*_*x*_, respectively. The occurrence frequency *f*_*x*_(*p*) of nucleotide *x* at the codon position *p* is defined as

$$f_{x}(p)=\sum_{p^{\prime }=0}^{\frac{N}{3}-1}u_{x}[3p^{\prime }+p],\;p=0,1,2.$$ (2)

From *f*_*x*_(*p*) of Eq (2), the TBP spectrum takes the form [18, 19]:

$$\begin{array}{l} P_{x}=S_{x}\left[N/3\right]\\ \text{ =}|\sum_{p^{\prime }=0}^{N/3-1}u_{x}[3p^{\prime }+0]e^{-i2\pi (3p^{\prime }+0)/3}+\sum_{p^{\prime }=0}^{N/3-1}u_{x}[3p^{\prime }+1]e^{-i2\pi (3p^{\prime }+1)/3}\\ \;{+\sum_{p^{\prime }=0}^{N/3-1}u_{x}[3p^{\prime }+2]e^{-i2\pi (3p^{\prime }+2)/3}|}^{2}\\ \;={\left|f(0)\cdot 1+f(1)\cdot e^{-i2\pi /3}+f(2)\cdot e^{-i4\pi /3}\right|}^{2}\\ \;={\left|f_{x}(0)\cdot 1+f_{x}(1)\cdot e^{-i2\pi /3}+f_{x}(2)\cdot e^{i2\pi /3}\right|}^{2}. \end{array}$$ (3)

### 2.2 Procedures of Singularity Detection

The singularities (or sharp variation points) often carry the most important information in the analysis of transient signal. Mallat's WTMM theory proves that the singularities of a signal can be measured from the evolution of the wavelet transform maxima across scales [44]. In this section, we first describe the implementation issues of the stationary wavelet transform (SWT) [54–57]. The second part of this section introduces the basic principles of the WTMM method. Then we give a fast reconstruction algorithm of wavelet coefficients from WTMM points, based on Hermite interpolation [58–59]. Finally, an optimized algorithm of singularity detection has been proposed for tracking WTMM and extracting useful information easily and correctly.

#### 2.2.1 SWT method

The SWT method having been independently discovered for different purposes and given a number of different names [54–57], including shift/translation invariant wavelet transform, undecimated discrete wavelet transform, or redundant wavelet transform. The key point is that it is redundant, shift invariant, and it gives a denser approximation to the continuous wavelet transform than the approximation provided by the orthonormal discrete wavelet transform (DWT). Let $h\in l^{2}(\mathbb{Z})$ and $g\in l^{2}(\mathbb{Z})$ be the scaling and wavelet filters of an orthonormal DWT, respectively. The scaling filter and wavelet filter of an SWT at scale *j* + 1 is defined recursively as

$$\begin{array}{l} {\bar{h}}_{j+1}[k]=h_{j}[k]↑2=\{\begin{array}{cc} h_{j}[k/2], & \text{if }k\text{ is even}\\ 0,\; & \text{if }k\text{ is odd} \end{array},\\ {\bar{g}}_{j+1}[k]=g_{j}[k]↑2=\{\begin{array}{cc} g_{j}[k/2], & \text{if }k\text{ is even}\\ 0,\; & \text{if }k\text{ is odd} \end{array}, \end{array}$$ (4)

where ${\bar{h}}_{0}[k]=h[k]$ and ${\bar{g}}_{0}[k]=g[k]$.

The SWT decompose a signal *v*_0_ into a coefficient set *C* = {*w*_1_, …,*w*_*J*_, *v*_*J*_} using an analysis filter bank $\left(\bar{h},\bar{g}\right)$ of Eq (4), where *w*_*j*_ is the wavelet coefficients at scale *j* and *v*_*J*_ is the scaling coefficients at the coarsest resolution. The passage from one resolution to the next one is obtained by

$$\begin{array}{l} v_{j+1}[k]={\bar{h}}_{j}[-k]*v_{j}[k],\\ w_{j+1}[k]={\bar{g}}_{j}[-k]*v_{j}[k], \end{array}$$ (5)

where *j* = 0, …*J*−1 and “*” denotes convolution.

The reconstruction of SWT is given by: $v_{j}[k]=\frac{1}{2}({\tilde{h}}_{j}[k]*v_{j+1}[k]+{\tilde{g}}_{j}[k]*w_{j+1}[k])$. The filter bank $\left(\bar{h},\bar{g},\tilde{h},\tilde{g}\right)$ needs to satisfy the exact reconstruction condition: $\bar{H}(z^{-1})\tilde{H}(z)+\bar{G}(z^{-1})\tilde{G}(z)=1$.

#### 2.2.2 Basic principles of WTMM

*Definition* [44]: Let *W*_*a*, *t*_ be the wavelet transform of a signal *s*(*t*) at the scale *a* and the position *t*.

- A WTMM is defined as a point (*a*_0_, *t*_0_) such that $\left|W_{a_{0},t}\right|<\left|W_{a_{0},t_{0}}\right|$ when *t* belongs to either a right or the left neighborhood of *t*, and $\left|W_{a_{0},t}\right|\leq \left|W_{a_{0},t_{0}}\right|$ when *t* belongs to the other side of the neighborhood of *t*_0_. We call maxima line, any connected curve in the scale space (*a*, *t*) along which all points are WTMM.
- For any point *t*_1_ ∈[*p*, *q*], *t*_1_ ≠ *t*_0_, *s*(*t*)is uniformly Lipschitz *n* in a neighborhood of *t*_1_. Let *α* < *n*, α ∉ ℤ, then the signal *s*(*t*) is Lipschitz *α* at *t*_0_, if and only if there exists a constant *A* at each modulus maxima (*a*, *t*) it holds that

$$\left|W_{a,t}\right|\leq Aa^{\alpha },\text{ i.e.},\text{ log}\left|W_{a,t}\right|\leq \text{log}(A)+\alpha \text{log}(a).$$ (6)

Let *a* = 2^*j*^, we can rewrite Eq (6) in the form: log|*W*_*j*, *t*_| ≤ log(*A*) + *αj*. Mallat has proved that the signal has singularities whose Lipschitz regularity are positive, the amplitudes of the WTMM would increase as *j* increases, while the noise creates singularities whose Lipschitz regularity is negative, its modulus maximum will decrease with the increasing of the level *j*.

#### 2.2.3 Reconstruction of wavelet coefficients using Hermite interpolation

As a method of piecewise-polynomial interpolation, Hermite interpolation matches the observed values of their first derivatives, which agrees well with the fact that the absolute value of the first derivative of a WTMM is zero. In [58] and [59], a Hermite interpolation polynomial is utilized to reconstruct wavelet coefficients from WTMM points, which yields to high computational efficiency compared with Mallat's alternating projection reconstruction algorithm. In this study, we use Hermite interpolation to accelerate the reconstruction process of wavelet coefficients. For simplicity, let *W*_*j*, *t*_ be the wavelet coefficients of a function at the position *t*, where *j* represents the resolution level. Let *A*_*j*, *r*_(*r* = 1, 2, …, *r*_0_) denote the local maxima of *W*_*j*, *t*_ for modulus maximum lines at the position *t*_*j*, *r*_, the reconstructed wavelet coefficients can be given as follows:

$${W^{\prime }}_{j,\;t}=A_{j,\;r}\left[1-2\frac{t-t_{j,\;r}}{t_{j,\;r}-t_{j,\;r+1}}\right]{\left[\frac{t-t_{j,\;r+1}}{t_{j,r}-t_{j,\;r+1}}\right]}^{2}+A_{j,\;r+1}\left[1-2\frac{t-t_{j,\;r+1}}{t_{j,\;r\text{+}1}-t_{j,\;r}}\right]{\left[\frac{t-t_{j,\;r}}{t_{j,\;r}-t_{j,\;r+1}}\right]}^{2},$$ (7)

where *t* ∈ [*t*_*j*, *r*_, *t*_*j*, *r+1*_] and *r* = 1, 2, …,*r*_0_−1. Eq (7) gives the wavelet coefficient points between adjacent local maxima at every resolution level. Note that the degree of the interpolation polynomial is only three. Therefore, this reconstruction scheme simplifies and accelerates the reconstruction process.

#### 2.2.4 An optimized algorithm for singularity detection with WTMM

In this subsection, we attempt to discriminate between the singular points of exons and introns, both presented in the DNA sequences. Given the input signal and the input noise (detailed in Section 2.3.2), our singularity detection algorithm for exon detection is described as follows:

1. Select the wavelet base and the maximum decomposition level *J*. Then, transform the input signal and the input noise into the coefficient set *C* = {*w*_1_, …, *w*_*J*_, *v*_*J*_} and the wavelet coefficient set $W^{\left(n\right)}=\left\{w_{1}^{\left(n\right)},\ldots ,w_{J}^{\left(n\right)}\right\}$, respectively, by the SWT method.
2. Calculate the local modulus maxima and their positions on each level from the wavelet coefficient set *W* = {*w*_1_, …, *w*_*J*_} of the input signal. At level *J*, retain the local modulus maxima which is greater than a threshold value and delete the local modulus maxima which is less than this threshold. In this paper, the threshold value is decided by
  $$Th=c\cdot \left(\text{max}(w_{J}^{\left(n\right)})/J\right),$$ (8)
  where *c* is the weight value determined by experience.
3. At level *j* = *J*−1, search modulus maxima that propagate from level *J* to *J*−1. Set *j* = *j*−1, repeat the process until *J* = 1. The maxima set for modulus maximum lines is marked as $W^{\prime }=\left\{{w^{\prime }}_{1},\ldots ,{w^{\prime }}_{J}\right\}$.
4. Utilize the Hermite interpolation polynomial method to reconstruct the wavelet coefficients from $W^{\prime }=\left\{{w^{\prime }}_{1},\ldots ,{w^{\prime }}_{J}\right\}$, then perform the inverse SWT to obtain the desired signal.

### 2.3 Method for Short Exon Detection Based on WTMM

In this section, we first describe a method to estimate the background noise of intron regions by using a notch filter with its notch centered at *ω*_0_ = 2*π*/3. The second part details the construction algorithm of nucleotide distribution sequence. Finally, we present our WTMM-based method for short exon detection.

#### 2.3.1 Estimate background noise using notch filter

**Fig 2:**
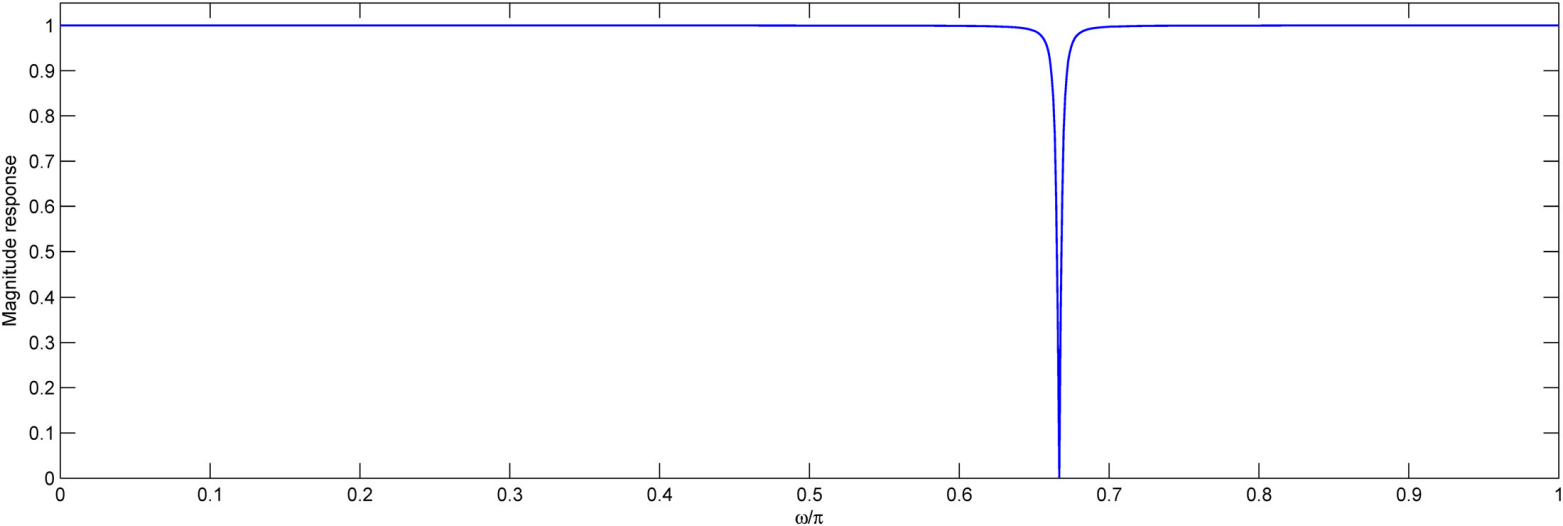
Notch filter responses for *R* = 0.992.

In order to estimate the noise level in intron regions, we suppress the TBP components from background information by filtering the genomic sequence through a notch filter with passband centered at *ω*_0_ = 2*π*/3. The notch filter can be obtained by starting from a second order all-pass filter [22]

$$A(z)=\frac{R^{2}-2R\text{cos}\theta z^{-1}+z^{-2}}{1-2R\text{cos}\theta z^{-1}+R^{2}z^{-2}},$$ (9)

which has poles at *Re*^±*jθ*^ and zeros at 1/*Re*^±*jθ*^. Then the notch filter *G*(*z*) has the form

$$G(z)=\frac{1+A(z)}{2}=K\left(\frac{R^{2}-2\text{cos}\omega_{0}z^{-1}+z^{-2}}{1-2R\text{cos}\theta z^{-1}+R^{2}z^{-2}}\right),$$ (10)

where *K* = (1 + *R*^2^)/2, cos*ω*_0_ = 2*R*cos*θ*/(1 + *R*^2^) and *R* = 0.992. By choosing *ω*_0_ = 2*π*/3, the filter *G*(*z*) can be used to suppress the TBP components of the DNA effectively [30]. The response of *G*(*z*) is shown in Fig 2.

#### 2.3.2 Construction algorithm of nucleotide distribution sequence

Set *N* and *L* to be positive integers of multiple 3. Let *u*_*x*_(*x* ∈ {*A*−*T*, *C*−*G*}) denote a DNA paired-numerical sequence of length *N*. The sequence of nucleotide distribution is calculated as follows:

1. Slice *u*_*x*_ into segments (or windows) of length *L* along the forward and reverse directions of the DNA sequence respectively, where neighboring segments overlap by *L* − 3 points. The *l*-th segment of *u*_*x*_ in both directions can be represented as
  $$D_{\kappa 1,\;x}(l)=\left\{u_{\kappa 1,\;x}[3l],u_{\kappa 1,\;x}[3l+1],\ldots ,u_{\kappa 1,\;x}[3l+L-1]\right\},\;l=0,1,\ldots ,L_{D}$$ (11)
  and
  $$D_{\kappa 2,\;x}(l)=\left\{u_{\kappa 2,\;x}[3(L_{D}-l)+L-1],u_{\kappa 2,\;x}[3(L_{D}-l)+L-2],\ldots ,u_{\kappa 2,\;x}[3(L_{D}-l)]\right\},\;l=0,1,\ldots ,L_{D},$$ (12)
  where *L*_*D*_ = (*N*−*L*)/3, *κ*1 and *κ*2 represent the forward and reverse directions of DNA sequence respectively.
2. Let *f*_*κ*, *x*_(*l*, *p*) be the occurrence frequencies of nucleotides at codon position *p* of *D*_*κ*, *x*_(*l*), where *κ* ∈ {*κ*1, *κ*2} and *p* ∈ {0, 1, 2}. The sequence of nucleotide distribution along one direction of the DNA sequence is defined as
  $$\begin{array}{cc} F_{\kappa ,\;x} & =\left\{{\bar{f}}_{\kappa ,\;x}(l,0),{\bar{f}}_{\kappa ,\;x}(l,1),{\bar{f}}_{\kappa ,\;x}(l,2)\right\}\\ & =\{{\bar{f}}_{\kappa ,\;x}(0,0),{\bar{f}}_{\kappa ,\;x}(0,1),{\bar{f}}_{\kappa ,\;x}(0,2),{\bar{f}}_{\kappa ,\;x}(1,0),{\bar{f}}_{\kappa ,\;x}(1,1),{\bar{f}}_{\kappa ,\;x}(1,2),\\ & \;\ldots ,{\bar{f}}_{\kappa ,\;x}(\left(N-L\right)/3,0),{\bar{f}}_{\kappa ,\;x}(\left(N-L\right)/3,1),{\bar{f}}_{\kappa ,\;x}(\left(N-L\right)/3,2)\}, \end{array}$$ (13)
  where ${\bar{f}}_{\kappa ,\;x}(l,p)=f_{\kappa ,\;x}(l,p)-\frac{1}{3}\sum_{p=0}^{2}f_{\kappa ,\;x}(l,p)$.
3. With the DNA sequence *u*_*x*_(*x* ∈ {*A*−*T*, *C*−*G*}) taken as input, let $u_{x}^{\left(n\right)}\;$ denote the output of the notch filter *G*(*z*) of Eq (10). Set $u_{x}=u_{x}^{\left(n\right)}$, and repeat the step (1) and step (2), the nucleotide distribution sequence for estimated noise is marked as
  $$F_{\kappa ,\;x}^{\left(n\right)}=\left\{{\bar{f}}_{\kappa ,\;x}^{\left(n\right)}(l,0),{\bar{f}}_{\kappa ,\;x}^{\left(n\right)}(l,1),{\bar{f}}_{\kappa ,\;x}^{\left(n\right)}(l,2)\right\}.$$ (14)

#### 2.3.3 Short exon detection algorithm based on WTMM

**Fig 3:**
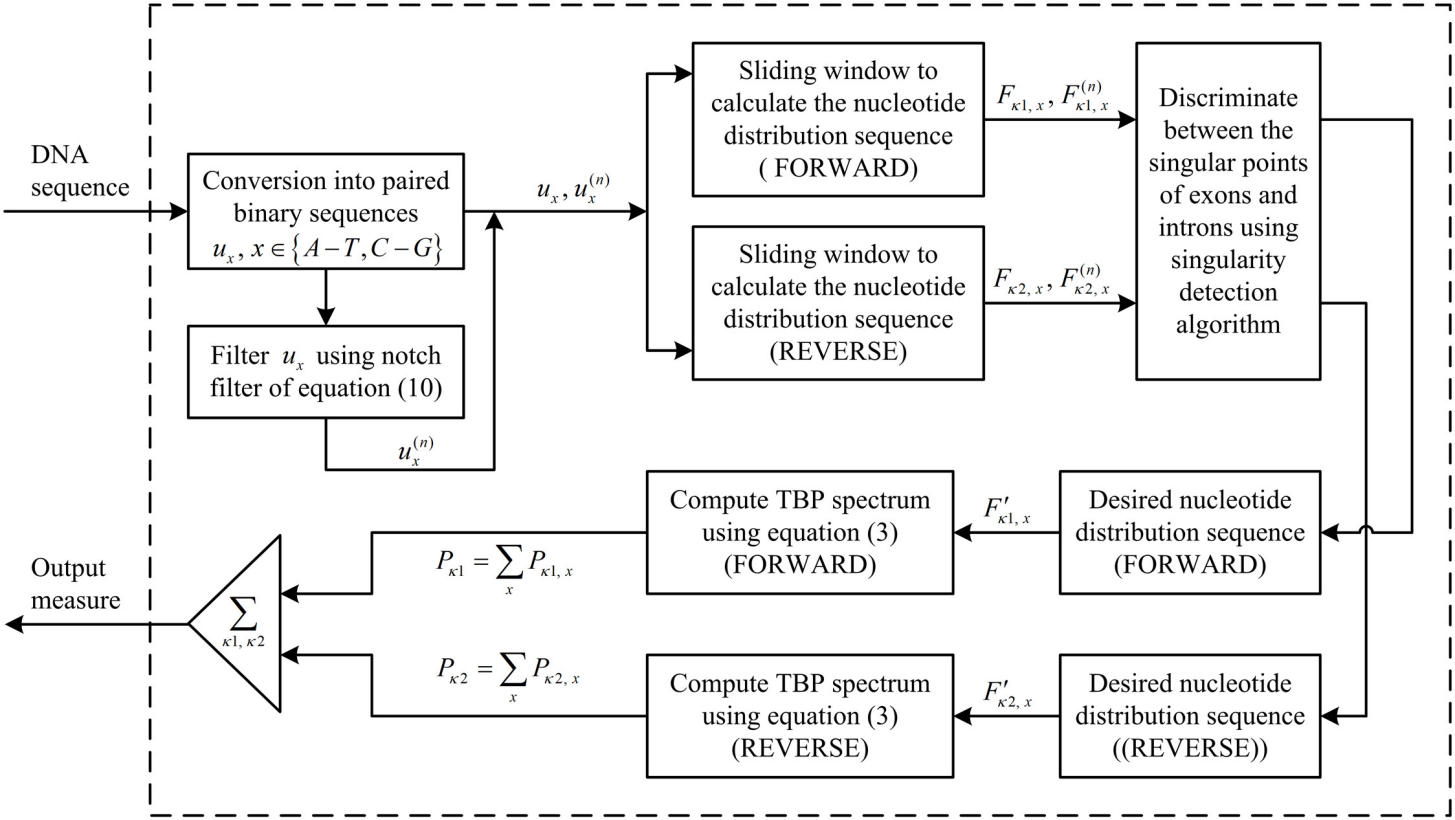
Flow chart of the WTMM method for exon detection.

Following the Section 2.2.4 and Section 2.3.2, our WTMM-based method for short exon detection works as follows, and the flow chart of this algorithm below is shown in Fig 3.

Step 1: Take *F*_*κ*, *x*_ and ${\bar{F}}_{\kappa ,x}^{\left(n\right)}$ as inputs, where *κ* ∈ {*κ*1, *κ*2} and *x* ∈ {*A*−*T*, *C*−*G*}. Then, perform the singularity detection algorithm on the inputs, to obtain the desired sequence
  $${F^{\prime }}_{\kappa ,\;x}=\left\{{f^{\prime }}_{\kappa ,\;x}(l,0),{f^{\prime }}_{\kappa ,\;x}(l,1),{f^{\prime }}_{\kappa ,\;x}(l,2)\right\},\;l=0,1,\ldots ,\left(N-L\right)/3.$$ (15)
Step 2: From Eqs (3) and (15), the TBP spectrum for the *l*-th segment in one direction is
  $$\begin{array}{l} P_{\kappa }\left(l\right)\text{= }\sum_{x}P_{\kappa ,\;x}\left(l\right)\\ \text{ =}\sum_{x}{\left|{f^{\prime }}_{\kappa ,\;x}(l,0)\cdot 1+{f^{\prime }}_{\kappa ,\;x}(l,1)\cdot e^{-i2\pi /3}+{f^{\prime }}_{\kappa ,\;x}(l,2)\cdot e^{i2\pi /3}\right|}^{2}. \end{array}$$ (16)
Step 3: In view of Eq (16), the output feature value can be calculated based on the following equation:
  $$P\left(l\right)\text{= }\sum_{\kappa }P_{\kappa }(l).$$ (17)
Step 4: Interpolate *P*(*l*) to get the length of the signal back to *N*.

## 3 Results and Discussion

### 3.1 General Setting

In this section, we provide experiments of exon detection by using the WTMM-based method. A window length of 90 points is used throughout the experiments, and the input is represented up to 4 resolution levels through the biorthogonal wavelet function Bior1.3. In our WTMM-based method, the moderate value of weight coefficient of Eq (8) is set to *c* ∈ [0.6, 0.7]. Herein, the WTMM-based method with weight coefficient *c* = 0.7 is denoted by WTMM I, while the WTMM II denotes the WTMM approach for the weight coefficient *c* = 0.6.

As a comparison, we have evaluated the general performances of three other methods: EPND [25], MGWT [26] and FFTEMD [27]. In the case of MGWT, the window length is 1200, and the scale parameter is set to 40 exponentially-separated values between 0.2 and 0.7 for four input-sequences. In order to investigate the performance of MGWT on short exon detection, the operation on MGWT with window length 1200 points and scale values exponentially separated between 0.1 and 0.7 is denoted by MGWT I, while MGWT II denotes the MGWT with window length 900 points and scale values exponentially separated between 0.2 and 0.7. To quantify the comparison, the magnitudes of TBP spectrums obtained from the WTMM I, WTMM II, MGWT, MGWT I, MGWT II and FFTEMD on each DNA sequence have been normalized with values between 0 and 1.

### 3.2 Data Resources

**Fig 4:**
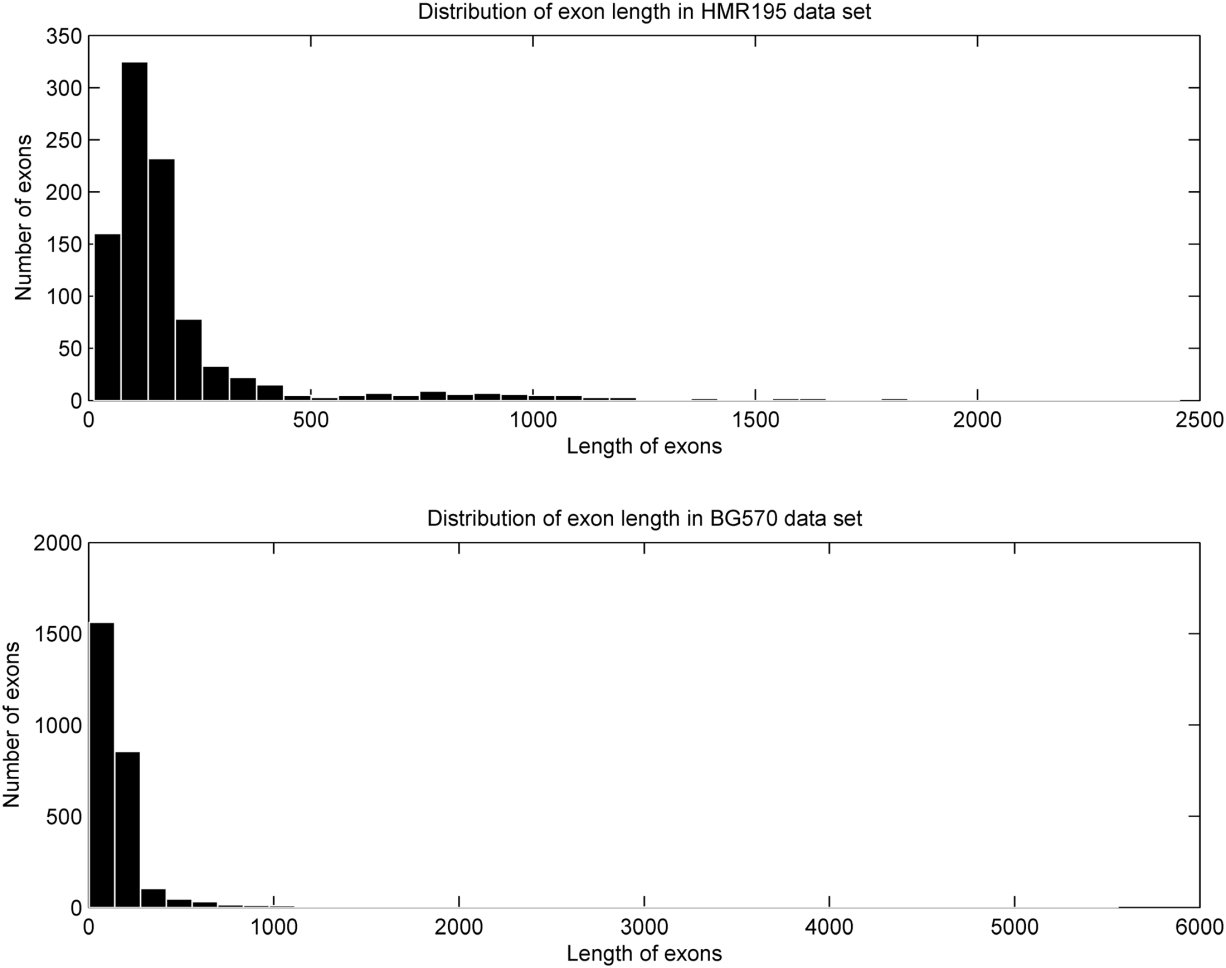
Distribution of exon lengths in two benchmark data sets.

To evaluate the performance of various methods, the DNA sequence AB021866 of H. sapien (GenBank file AB021866) is used in our study. In order to comprehensively evaluate and compare our WTMM-based algorithm with the other methods, the two benchmark data sets HMR195 [60] and BG570 [61] have been considered. Table 2 summarizes the statistics of the considered test data sets. Fig 4 shows the distribution of the exon lengths in these two benchmark data sets.

**Table 2:** Statistics of the test data sets.

Dataset	Species	Genes	Length	Exons	Average length of exons	Proportion of exons	Exons (≤150 bp) density in total exons
HMR195	Mammalian	195	1,383,720	948	208	14%	58.97%
BG570	Vertebrate	570	2,892,149	2,649	168	15.37%	62.67%

### 3.3. Evaluation Metrics

In order to evaluate the performance of considered methods, we compute the following counts: *TP* is the true positive, which is the length of nucleotides of correctly detected exons; *TN* is the true negative, which is the length of nucleotides of correctly detected introns; *FN* is the false negative, which is the length of nucleotides of wrongly detected introns; and *FP* is the false positive, which is the length of nucleotides of wrongly detected exons. Following the evaluation metric included in [60], the performances of various methods are measured in terms of the correlation coefficient (CC)

$$CC=\frac{TP\times TN-FP\times FN}{\sqrt{\left(TP+FP)(TP+FN)(TN+FP)(TN+FN\right)}},$$ (18)

which provides a measure of overall detection accuracy.

We also calculate the sensitivity *Sn* and specificity *Sp*:

$$Sn=\frac{TP}{TP+FN},$$ (19)

$$Sp=\frac{TN}{TN+FP}.$$ (20)

The sensitivity provides a measure of the proportion of exon nucleotides that have been correctly detected as exons, and the specificity provides the proportion of intron nucleotides that have been correctly detected as introns. We use the average value of *Sn* and *Sp* as a measure of the probability of correct detection.

For validation of the classification of sequences, we employ the receiver operating characteristic (ROC) curve [62, 63], which explores the effects on *Sensitivity* and 1 − *Specificity*.

### 3.4 Performance Evaluation on a Typical Sequence

**Fig 5:**
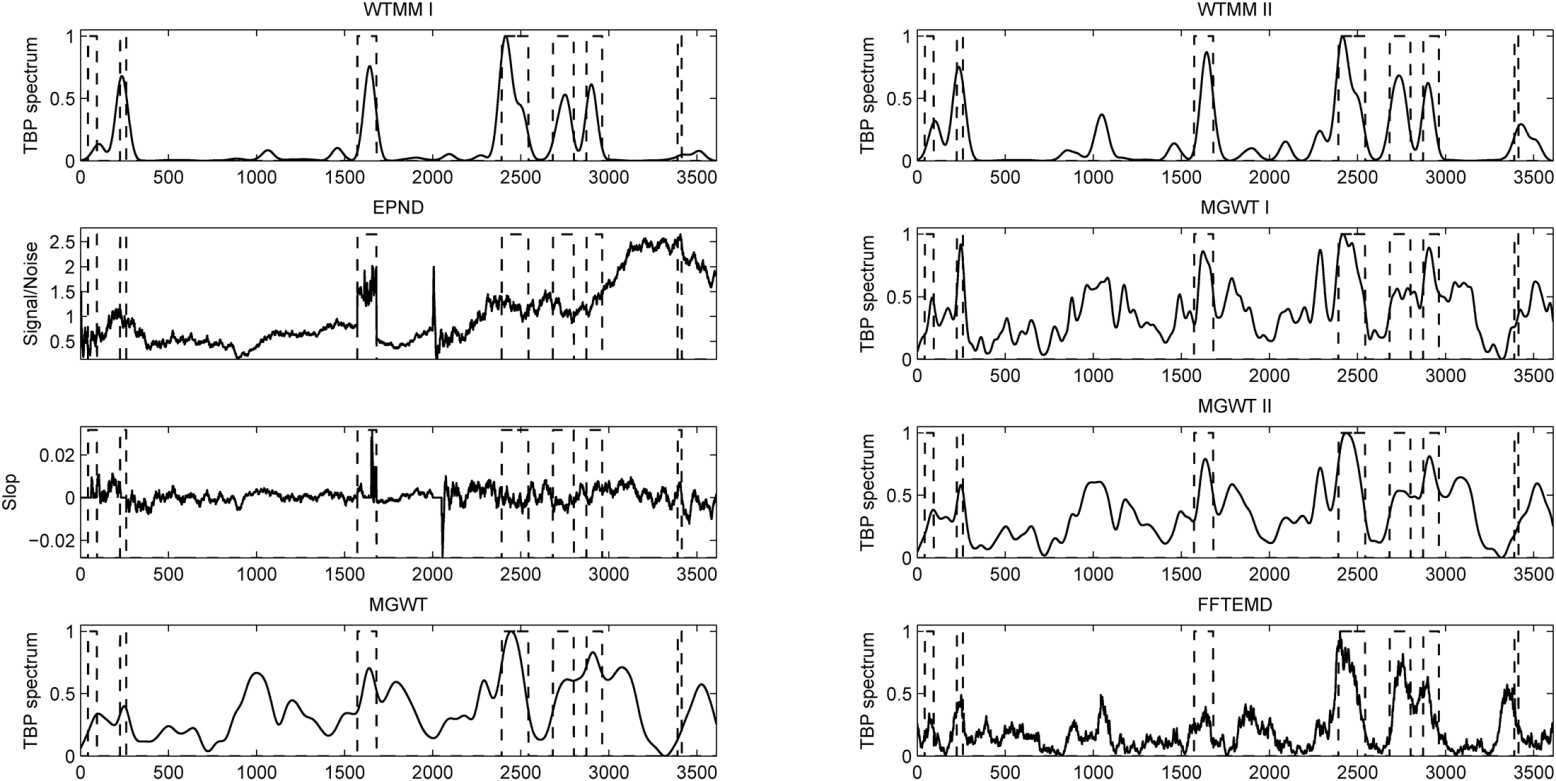
Detection plots for sequence AB021866 using various methods.

**Fig 6:**
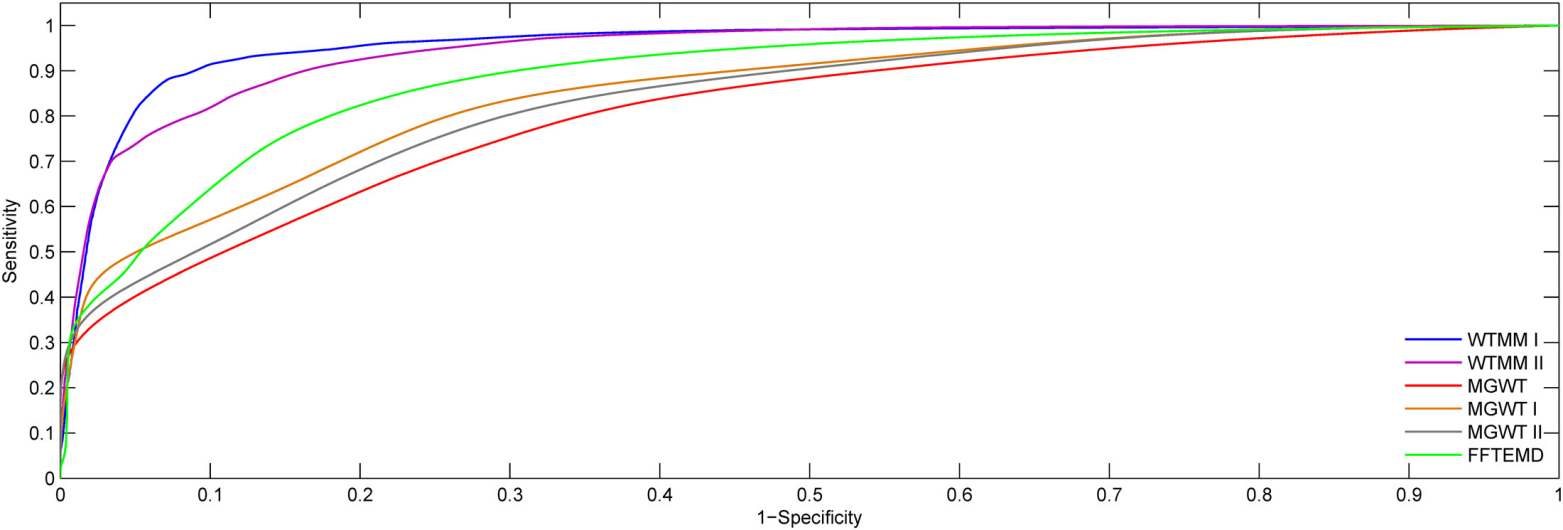
ROC curves for performance evaluation using the sequence AB021866.

The DNA sequence AB021866 of 3,611 bp contains seven exons located at relative positions of 43–93, 225–259, 1,573–1,681, 2,393–2,543, 2,683–2,801, 2,874–2,962 and 3,392–3,413. The experimental results obtained from consider methods on this particular sequence are presented in Fig 5. Abscissa axes of all the plots represent the relative base positions of nucleotides on the DNA sequence, and the actual locations of exons are marked with rectangles in dashed line. From Fig 5, the seven short exons are distinct in the results of WTMM I and WTMM II. The CC values obtained from the WTMM I, WTMM II, EPND, MGWT, MGWT I, MGWT II and FFTEMD on this particular sequence are 0.814, 0.740, 0.457, 0.543, 0.505 and 0.625, respectively. The ROC curves achieved in the WTMM I, WTMM II, MGWT, MGWT I, MGWT II and FFTEMD methods for this sequence are shown in Fig 6. This figure clearly indicates that our two WTMM methods outperform their counterparts along the ROC curves at all classification thresholds.

### 3.5 Performance Evaluation on Benchmark Data Sets

To evaluate the performances of the considered methods over a larger number of sequences, we carry the classification experiments to compare the efficiencies of the introduced methodologies over HMR195 and BG570 data sets.

**Fig 7:**
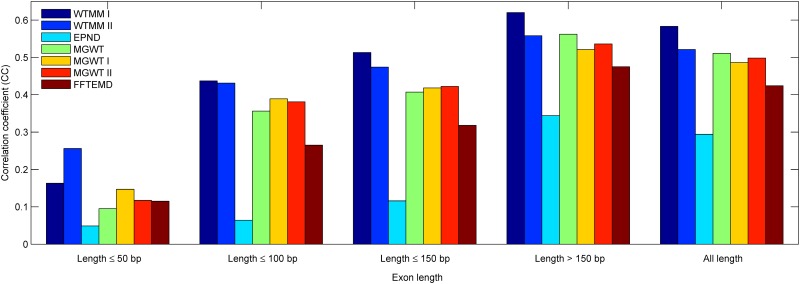
The bar chart of CC of various methods over HMR195&BG570 when they have been applied to sequences of five ranges of exon lengths.

**Fig 8:**
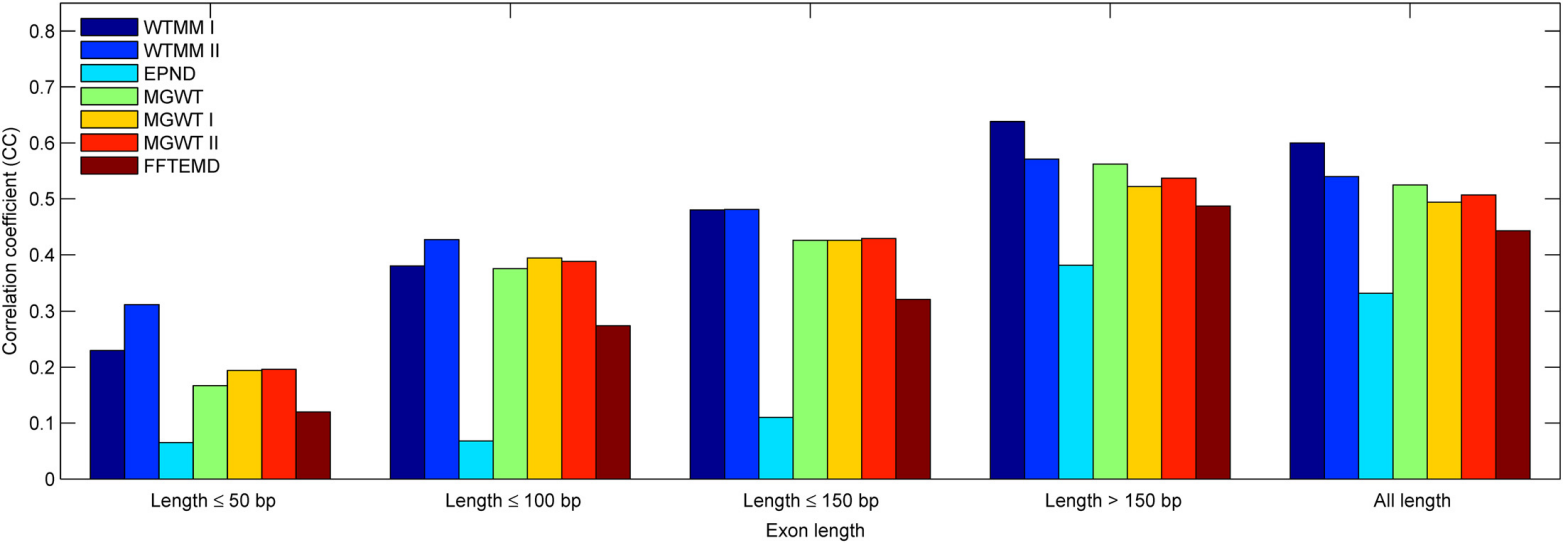
The bar chart of CC of various methods over HMR195 when they have been applied to sequences of five ranges of exon lengths.

**Fig 9:**
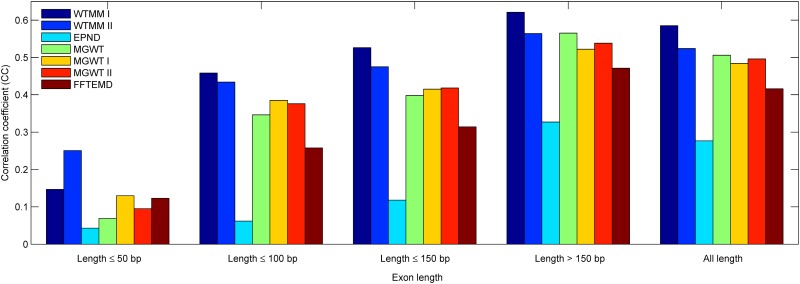
The bar chart of CC of various methods over BG570 when they have been applied to sequences of five ranges of exon lengths.

In order to set up a comprehensive comparison for exon detection, especially short exon detection, we first conduct an experiment to calculate the CC values achieved in considered methods over exons in five ranges of lengths (length ≤50 bp, length ≤100 bp, length ≤150 bp, length >150 bp and all length). In our view, the exons with these ranges are relatively short (≤150 bp) and long (>150 bp). Fig 7 depicts the best accuracy in terms of the correlation coefficient calculated at each group of exons from the HMR195&BG570. Similar results are shown in Figs 8 and 9 corresponding to the HMR195 and BG570, respectively. The results in Fig 7 show that the WTMM I outperforms the performance of the other consider methods at these five ranges of exon lengths. The WTMM II gives good results at the first three ranges and is close to the MGWT at the range >150, while it slightly exceeds the other methods at the range ≤50. In addition, the performances of the MGWT I and MGWT II outperform the MGWT at ranges ≤50, ≤100 and ≤150, but less than the MGWT at the ranges of >150 and all length. In general, our WTMM-based method consistently generates higher detection accuracy when it deals with exons that are either relatively short or long in length.

Table 3 summaries the experiment results of the different considered methods at each group of exons from the HMR195&BG570 in terms of the correlation coefficient. Similar results are listed in Tables 4 and 5 corresponding to the HMR195 and BG570, respectively. By comparison with the existing methods, the detection results of Table 3 show that: 1) Our WTMM-based method exhibits at least improvement of 74.2%, 12.3% and 21.6% on the exons of lengths not greater than 50 bp, 100 bp and 150 bp, respectively; 2) The WTMM-based method reveals at least improvements of 10.3% and 14.1% over the exons of lengths greater than 150 bp and all length, respectively.

**Table 3:** Summary of the experiment results of the different considered methods at each group of exons from HMR195&BG570.

Method	Correlation coefficient (CC)				
	≤50 bp	≤100 bp	≤150 bp	>150 bp	All length
WTMM I	0.163	0.437	0.513	0.620	0.583
WTMM II	0.256	0.431	0.474	0.558	0.521
EPND	0.049	0.064	0.116	0.344	0.294
MGWT	0.095	0.356	0.407	0.562	0.511
MGWT I	0.147	0.389	0.418	0.521	0.486
MGWT II	0.117	0.381	0.422	0.536	0.498
FFTEMD	0.115	0.265	0.318	0.475	0.424

**Table 4:** Summary of the experiment results of the different considered methods at each group of exons from HMR195.

Method	Correlation coefficient (CC)				
	≤50 bp	≤100 bp	≤150 bp	>150 bp	All length
WTMM I	0.230	0.380	0.480	0.638	0.600
WTMM II	0.312	0.427	0.481	0.571	0.540
EPND	0.065	0.068	0.110	0.381	0.332
MGWT	0.167	0.375	0.426	0.562	0.525
MGWT I	0.194	0.394	0.426	0.522	0.494
MGWT II	0.196	0.388	0.429	0.537	0.507
FFTEMD	0.120	0.274	0.321	0.487	0.443

**Table 5:** Summary of the experiment results of the different considered methods at each group of exons from BG570.

Method	Correlation coefficient (CC)				
	≤50 bp	≤100 bp	≤150 bp	>150 bp	All length
WTMM I	0.147	0.458	0.526	0.621	0.585
WTMM II	0.251	0.434	0.475	0.564	0.524
EPND	0.043	0.062	0.118	0.327	0.277
MGWT	0.069	0.346	0.398	0.565	0.506
MGWT I	0.130	0.385	0.415	0.522	0.484
MGWT II	0.095	0.376	0.418	0.538	0.496
FFTEMD	0.123	0.258	0.314	0.471	0.416

**Fig 10:**
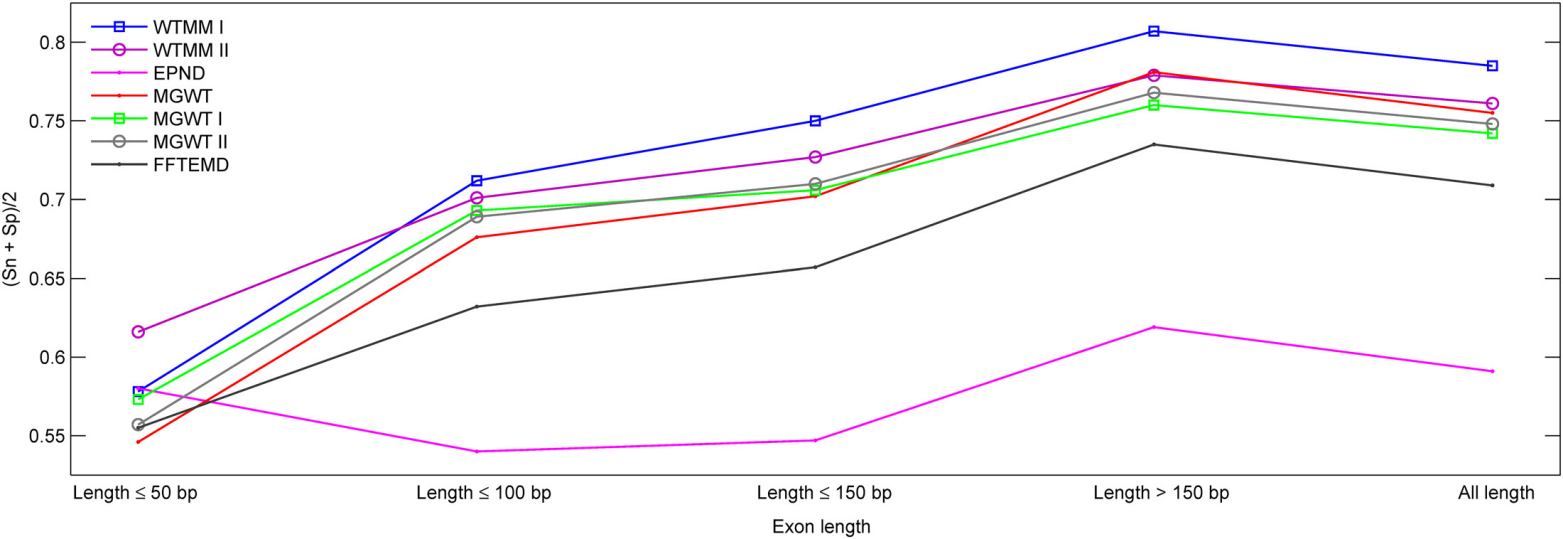
The plots of (*Sn* + *Sp*)/2 of various methods over HMR195&BG570 when they have been applied to sequences of five ranges of exon lengths.

An additional classification experiment is designed to assess the performances of introduced methods. Fig 10 depicts the best accuracy in terms of the average of *Sn* and *Sp* calculated at each group of exons from the HMR195&BG570. It should be noted that the EPND generates high accuracy at the range ≤50, which can be contributed to the statement [25]: “*If a DNA region less than 50 base pairs is identified as an intron*, *and is flanked by two exon regions*, *this region is often a false negative*, *and is reset as exon region; similarly*, *if a DNA region less than 50 base pairs is identified as an exon*, *and is flanked by two intron regions*, *this region is often a false positive*, *and is reset as an intron region*.”. These plots again establish the superiority of our WTMM-based algorithm over other methods in short exon detection.

**Fig 11:**
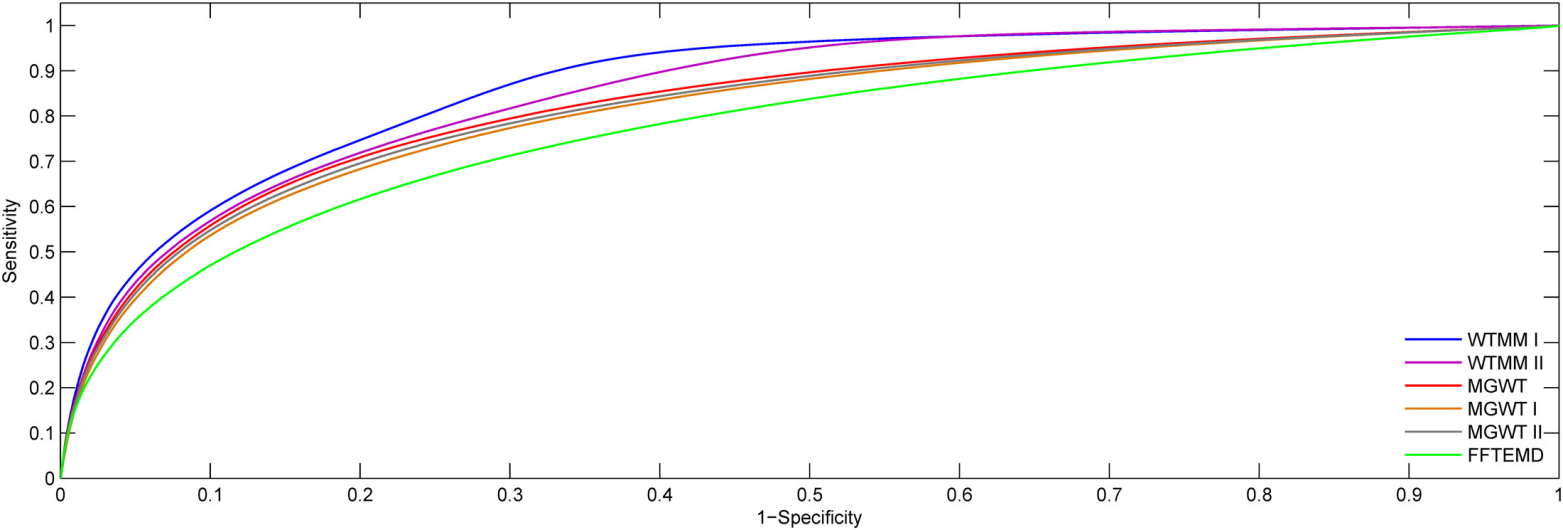
ROC plots for the WTMM I, WTMM II, MGWT, MGWT I, MGWT II and FFTEMD using HMR195&BG570.

The ROC curves obtained from the WTMM I, WTMM II, MGWT, MGWT I, MGWT II and FFTEMD methods for HMR195&BG570 are shown in Fig 11. It can be observed that the WTMM-based method generates higher detection accuracy compared with its counterparts. The experiments in ROC plots are consistent with the results of Figs 7 and 10.

### 3.6 Summary

**Fig 12:**
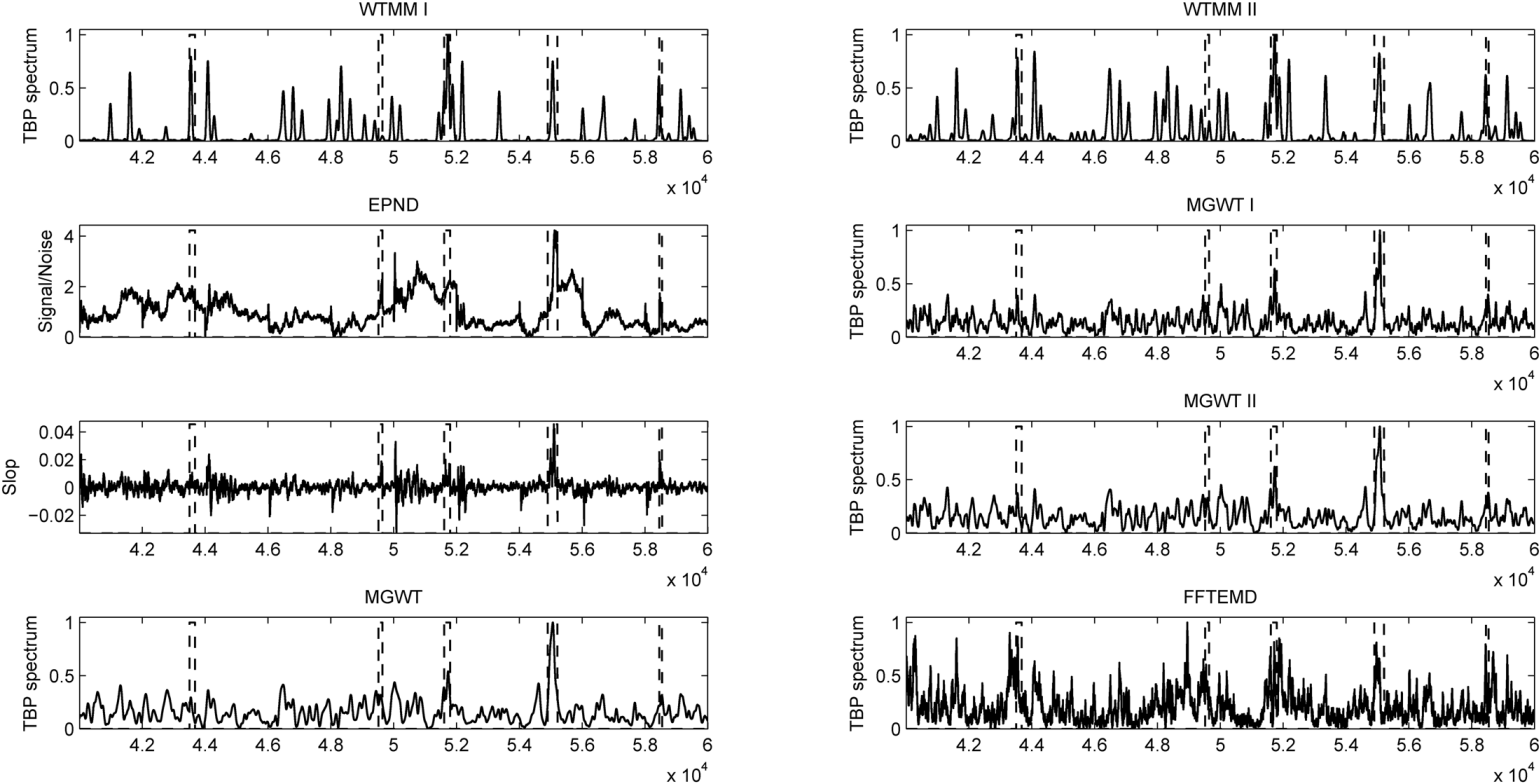
Detection plots for sequence BRCA1, in the range of 40,001–60,000 bp.

This research is aimed at detecting short exons in eukaryotic DNA sequences. The misinterpretation of exon locations will lead to a misunderstanding of the gene or exon properties. This open problem poses a challenge to understand and define precisely the biochemical processes and information involved in the pathway from DNA to proteins. Consequently, knowledge pertaining to exon locations may result in the design of customized drugs and new cures for diseases [1]. Fig 12 is an example of the detection plots for gene BRCA1 (GenBank file AY365046), in the range of 40,001–60,000 bp. This particular segment has five exons located at relative positions of 43,505–43,676, 49,518–49,644, 51,608–51,798, 54,905–55,215 and 58,458–58,545, corresponding to the exons 11–15 of BRCA1. Abscissa axes of all the plots represent the relative base positions, and the actual locations of exons are marked with rectangles (dashed line). In gene BRCA1, the mutations in exon 11 are associated with the forms of breast cancer [34–37]. It is clear that our WTMM-based algorithm can generate high peaks in the region of exon 11 than the other methods.

To summarize, we have used the WTMM technique to detect exons in eukaryotic genome sequences. Compared with three existing algorithms, the technique described in this paper provides an improvement in short exon detection. The WTMM-based method introduced in this study has the following three features: (1) This technique first constructs a sequence of nucleotide distribution to measure the 3-base periodicity. The spectral contents are calculated from the nucleotide frequencies in the three reading frames, which incorporates the reality of proteins' prefer specific amino acid compositions and reduces the computational cost. (2) The important feature of our method is to explore the evolution of singularities of short exons across scales from the local maxima of their wavelet transform modulus. Unlike the description of the overall regularity for exons, the proposed measure provides significant patterns that are rarely observed with the traditional methods. (3) The method calculates the output values from a paired-numerical representation in both forward and reverse directions, which reflects the reality of the structure of DNA and increases computational efficiency.

## 4 Conclusion

In this paper, a model-independent method based on the singularity detection with wavelet transform modulus maxima has been developed for detecting eukaryotic exons, especially short exons. The WTMM technique employed in our method makes it capable of working with short exon detection in the performance of experimental evaluation. The analysis of the proposed method has shown that it has an adaptive response to the location and spatial distribution of singular points represented by short exons. Experimental results show that the WTMM-based method outperforms the assessed model-independent methods for short exon detection in terms of evaluation metrics.
